# Zika Infection Disrupts Proteins Involved in the Neurosensory System

**DOI:** 10.3389/fcell.2020.00571

**Published:** 2020-07-29

**Authors:** Kathleen K. M. Glover, Ali Zahedi-Amiri, Ying Lao, Victor Spicer, Thomas Klonisch, Kevin M. Coombs

**Affiliations:** ^1^Department of Medical Microbiology and Infectious Diseases, University of Manitoba, Winnipeg, MB, Canada; ^2^Manitoba Centre for Proteomics and Systems Biology, Winnipeg, MB, Canada; ^3^Department of Human Anatomy and Cell Science, University of Manitoba, Winnipeg, MB, Canada; ^4^John Buhler Research Centre, Children’s Hospital Research Institute of Manitoba, Winnipeg, MB, Canada

**Keywords:** Zika virus, neurosensory alterations, proteomics, TMT mass spectrometry, neurodevelopment

## Abstract

Newly re-emerging viruses are of great global concern, especially when there are no therapeutic interventions available during the time of an outbreak. There are still no therapeutic interventions for the prevention of Zika virus (ZIKV) infections despite its resurgence more than a decade ago. Newborns infected with ZIKV suffer from microcephaly and delayed neurodevelopment, but the underlying causes are largely unknown. All viruses hijack the host cellular machinery to undergo successful replication. Our tandem mass tag mass spectrometry-based proteomic monitoring of cells infected with ZIKV revealed that among the thousands of host proteins dysregulated over time, many protein candidates were linked to neurodevelopmental processes, including the development of the auditory and visual/retinal system. The role of these dysregulated neurodevelopmental-associated host proteins for ZIKV propagation in eukaryotic cells remains elusive. For the first time, we present temporal neurodevelopmental proteomic responses in cells undergoing ZIKV infection. The future goal is to identify host proteins whose dysregulation results in neurosensory alterations reported in children born to ZIKV-infected mothers.

## Significance of Study

Therapeutic modalities for the management of ZIKV infections are still not available, and infected patients rely on supportive symptomatic treatment. Currently, no studies exist that investigate the role of ZIKV at the proteomic level on neuro-developmental defects, such as retinal disease or hearing loss. Our present study identified promising new host proteins with known functions in the neurosensory system that are altered by ZIKV. Such proteins may be suitable leads to better understand the detrimental neurodevelopmental outcomes on *in utero* ZIKV infection.

## Introduction

Zika virus (ZIKV), an arthropod-borne virus, has been known for decades, but useful therapeutic interventions are still lacking. There were global concerns when a high prevalence of ZIKV infections was reported in numerous countries in the early 2000s (Brasil et al., 2016). RNA nucleic acid testing is currently used to confirm ZIKV infections in suspected infected patients. Pregnant women are at high risk since the virus can cross the placenta and infect the developing fetus. ZIKV transplacental transmission was confirmed by detecting viral proteins and RNA in placental tissue samples from expectant mothers infected at different stages during pregnancy (Calvet et al., 2016; Martines et al., 2016; Sarno et al., 2016). Infection of the developing fetus leads to congenital abnormalities collectively known as Congenital Zika Syndrome (CZS) (Moore et al., 2017). CZS is characterized by a spectrum of neurological disorders in infants ranging from mild to severe brain damage with or without microcephaly, to severe forms that result in intrauterine death (Besnard et al., 2016; Schuler-Faccini et al., 2016).

Children born to ZIKV-infected mothers experience various developmental delays, the severity of which is assessed by the Bayley Scales of Infant and Toddler Development, 3rd edition (Bayley-III) (Lopes Moreira et al., 2018; Madaschi et al., 2016). These developmental challenges are attributed to delayed neurodevelopment and neurosensory alterations in longitudinal studies conducted on ZIKV-infected babies (Lopes Moreira et al., 2018). The Bayley-III uses scales to determine a series of emotional, cognitive, physical, and social developmental competencies of infants and toddlers to assess age-normalized abnormal development and to predict future challenges of these children when they attain school age. However, normal Bayley-III scores and CT scans do not guarantee normal development in some ZIKV-infected cohorts (Nielsen-Saines et al., 2019).

Identification of prognostic protein or gene markers for various disease conditions have been successfully used for the development of rapid diagnostic kits or vaccines, and have facilitated better understanding of underlying biological processes. We have used mass spectrometry (MS)-based proteomics to identify host proteins whose expression profiles were altered during ZIKV infection. In the present study, we used proteomic analyses of ZIKV-infected Vero cells and identified more than 6,000 host proteins at each of three time points investigated (12, 24, and 48 h after infection); 74 of these host proteins were significantly dysregulated by ≥ 2.0-fold. Expression dysregulation was validated by Western blot for five host proteins. Bioinformatic analyses predicted several pathways that were either activated or inhibited by ZIKV, including several linked to neurodevelopment and sensory functions.

## Materials and Methods

### Cells

Vero cells (ATCC^®^ Number: CCL-81^TM^) were maintained in Dulbecco’s modified Eagle’s medium (DMEM) supplemented with 10% Fetal Bovine Serum (FBS), 2 mM L-glutamine, non-essential amino acids, and sodium pyruvate at 37C in 5% CO_2_. After the third passage, cells grown to 60% confluency were infected with ZIKV.

### Infection

Vero cells were infected at a multiplicity of infection (MOI) of 3 plaque forming units (PFU) per cell to ensure >95% synchronous infection. Virus was allowed to adsorb for 2 h before DMEM supplemented with 2.5% FBS was added. Mock and time-matched ZIKV-infected cells were harvested at 12, 24, and 48 h post-infection (hpi), and proteomic analyses were performed on cell lysates obtained from three separate biological replicates.

### Protein Quantification

Individual time-matched Mock and ZIKV-infected cells were harvested using sterile scrapers. Cells were pelleted by centrifugation at 600 × *g* for 8 min and washed 3 × with sterile 1 × PBS. Washed cells were lysed with 4% SDS in 100 mM HEPES buffer pH 8.5. Cell lysates were centrifuged at 14,000 × *g* for 15 min at 11°C to remove insoluble cellular components. Total lysate protein concentrations were determined using a commercial Bradford total protein estimation method (Pierce Biotechnology, Rockford, IL, United States).

### Immunoblotting

To verify the infection status of cells, Western blot analyses were performed to probe for ZIKV non-structural protein NS1 (BioFront Cat. # BF-1225). Proteins from ZIKV-infected and mock-infected samples were separated by SDS-PAGE and transferred to 0.2 μm nitrocellulose membranes. Membranes were blocked in 5% skim milk for 1 h and incubated for 90 min with an antiserum against viral NS1 protein. Detection of ß-actin (Cell Signaling, Cat. # MABT144) served to estimate equal amounts of protein loaded and to normalize expression intensities of proteins. Selected dysregulated host proteins also were examined to verify mass spectrometry (MS) data. The proteins detected included SPARC (Cell Signaling, Cat. # 5420), BAD (Cell Signaling, Cat. # 9292), NF-κB2 (Cell Signaling, Cat. # 4882), DDX5 (Cell Signaling, Cat. # 9877), and EIF4A1 (Cell Signaling, Cat. # 9292). Primary antibody reactivity was detected with HRP-conjugated secondary antibodies (Cell Signaling).

### Tandem Mass Tags Mass Spectrometry Analyses

Quantified proteins from 12, 24, and 48 hpi samples were digested into peptides using the SP3 (single-pot solid-phase-enhanced sample preparation) procedure described by Sielaff (Sielaff et al., 2017). Briefly, proteins were trypsin digested for 14 h at 37°C and peptides were eluted. Tandem mass tags (TMT) labeling was performed as specified by the manufacturer (Thermo Fisher Scientific), except that TMT labels were dissolved in DMSO. Individual 6-plex TMT labeling was performed on 80 μg of each of three replicates of mock and three replicates of infected at each time point. Equivalent amounts of labeled samples within each TMT time set were mixed prior to 2D LC/MS/MS.

An Agilent 1100 series LC system with UV detector (214 nm) and 1mm × 100mm XTerra C18, 5 μm column (Waters, Ireland) was used for pH 10 first dimension reversed-phase separation. A gradient of 1.80% acetonitrile per minute (0.1–59.9% acetonitrile in 30 min) was delivered at a flow rate of 150 μL/min. Both eluents A (water) and B (1:9 water:acetonitrile) contained 20 mM ammonium formate at pH 10. Twenty 1-min fractions were collected and concatenated into 10 (#1 mixed with # 11, etc.) to provide optimal orthogonal separation. These fractions were lyophilized and resuspended in 0.1% formic acid for the second dimension analyses.

Analyses of TMT-labeled peptides were performed on an Orbitrap Q Exactive HF-X instrument (Thermo Fisher Scientific, Bremen, Germany). The sample was introduced using an Easy-nLC 1000 system (Thermo Fisher Scientific) at 1μg per injection. Mobile phase A was 0.1% (v/v) formic acid and mobile phase B was 0.1% (v/v) formic acid in 80% acetonitrile (LC-MS grade). Gradient separation of peptides was performed on a C18 (Luna C18; Calvet et al., 2016, 3 μm particle size [Phenomenex, Torrance, CA]) column packed in-house in Pico-Frit (100 μm × 30 cm) capillaries (New Objective, Woburn, MA, United States). Peptides were separated by the following gradient: 5% phase B over 2 min, 5–7% increase of phase B over 2 min, 7–25% over 60 min, 25–60% over 15 min, 60–90% over 1 min, with a final elution of 90% B for 10 min at a flow rate of 300 nL/min.

Data acquisition on the Orbitrap Q Exactive HF-X instrument was configured for data-dependent method using the full MS/DD-MS/MS setup in a positive mode. Spray voltage was set to 1.85 kV, funnel RF level at 40, and heated capillary at 275°C. Survey scans covering the mass range of 350–1500 m/z were acquired at a resolution of 120,000 (at m/z 200), with a maximum ion injection time of 60 ms, and an automatic gain control (AGC) target value of 3e6. For MS2 scan triggering, up to 20 of the most abundant ions were selected for fragmentation at 32% normalized collision energy, with intensity threshold kept at 6.3e4. AGC target values for fragment spectra were set at 1E5, which were acquired at a resolution of 30,000, with a maximum ion injection time of 80 ms and an isolation width set at 1.2 m/z. Dynamic exclusion of previously selected masses was enabled for 20 s, charge state filtering was limited to 2–6, peptide match was set to preferred, and isotope exclusion was on.

### Peptide and Protein Identification and Quantification

A database of protein sequences for ZIKV (Thai strain) and human (Uniprot 2016) was used for peptide/protein identification. For each time point every 1D LC-MS run in the 2D-LC-MS experiment was converted into an MGF file using the Proteome Discoverer bundled tool, as we have previously described (Glover et al., 2019; Honein et al., 2017; Scherer et al., 1995; Sher et al., 2019). These were then concatenated into a single MGF per time point. These three concatenated MFGs files were each searched against the database using X!tandem (cyclone 2012.10.01.1). Spectra files in MGF format, the peptide identification results, and the overall protein expression matrix are stored under accession MSV000085057 at the UCSD Centre for Computational Mass Spectrometry repository (https://massive.ucsd.edu).

Standard peptide identification settings were used: single missed cleavage tryptic peptides were permitted, with a parent and fragment mass tolerance of 10 ppm. A fixed post-translational modification of C + 57.021 was applied, and variable PTMs including N-terminal acetylation, deamidation, phosphorylation, and oxidation were permitted. Peptide assignment into source proteins was managed by X!tandem.

Peptide level TMT6 reporter tag intensities were integrated across a window of ±3 mDa each and corrected for isotopic overlap between channels using the supplied batch-specific correction matrix. Protein quantitation required at least two unique peptides with expectation values log(e) ≤ −1.5 each, yielding highly confident protein assignments of at least log(e) ≤ −3. The sum of peptide level TMT6 reporter tag intensities for each protein was converted into a log_2_ scale for simplified differential analysis.

### Statistical and Bioinformatics Analysis

Individual log_2_ protein differences between non-infected and each time-matched infected sample were converted to fold changes and *p*-values were calculated using Student’s *T*-test for grouped data to determine the level of significance. *Z*-scores for each replicate across all time points were calculated to additionally classify proteins that were not considered significant by *T*-test. A *p*-value of < 0.05 was used to select significantly dysregulated proteins and *Z*-score values of ≥+1.96σ and ≤−1.96σ were considered valid criteria for up- and down-regulation.

Time-point-specific datasets containing protein IDs, fold changes compared to mock-infected cells, and *p*-values were uploaded into and analyzed by Ingenuity Pathway Analysis (IPA) software. The IPA database was used to classify all significantly regulated proteins based on protein type and subcellular location. Graphic representation of the distribution of molecules in different cellular compartments was done by manually modifying cell graphic vector downloaded from the IPA pathway designer tool. Information regarding the top affected bio-functions, canonical pathways, upstream molecules, and interconnecting networks was exported by performing a core analysis in IPA. The STRING biological online database was used to obtain protein-protein interaction networks for significantly dysregulated proteins across the first two time points and for altered proteins corresponding to the neurosensory system at 48 hpi. Heatmaps were generated using MORPHEUS (Broad Institute, Cambridge, MA, United States) free online software by uploading protein IDs and fold changes. Representation of all graphs and volcano plots were performed using GraphPad Prism 6.0 software. Graphic components were designed and developed in Microsoft PowerPoint. ImageJ 1.51K was used to quantify band intensities from Western blot validations and statistical differences were assessed by one-way or two-way ANOVA (± SDE, *p*-values < 0.05).

## Results

### ZIKV Causes Specific Temporal Changes in the Cellular Proteome

**FIGURE 1:**
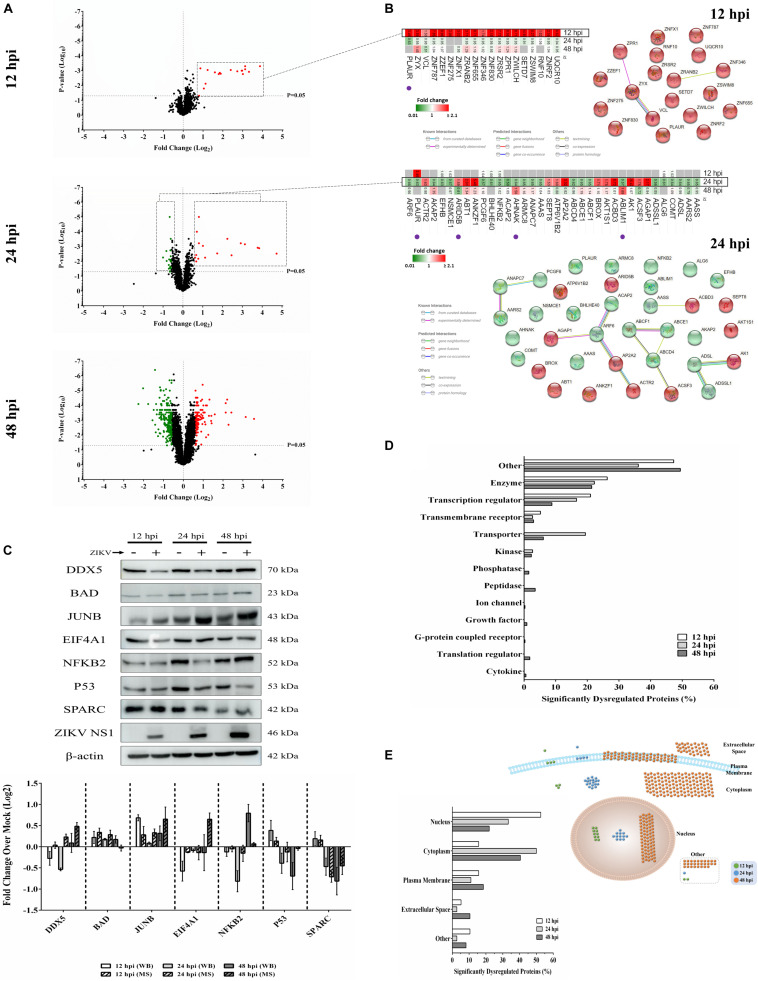
Mass spectrometry proteomic analysis of ZIKV-infected Vero cells. **(A)** Volcano plots displaying ZIKV-induced log_2_–fold protein level changes and significance of differentially regulated proteins at 12 (upper panel), 24 (middle panel), and 48 (lower panel) hours post-infection (hpi) (*p*-value < 0.05). **(B)** The quantitative comparison heatmaps indicating fold changes and protein-protein interaction networks of differentially dysregulated proteins across two time points (12 and 24 hpi). The protein-protein interaction network of the large number of differentially dysregulated proteins at 48 hpi is shown in Supplementary Figure S1. Proteins labeled with purple dots below heatmaps were significantly differentially expressed in the opposite direction at either earlier or later time points. **(C)** Western blot validation of selected differentially dysregulated proteins from mass spectrometry results. Representative blots are shown. Different loading controls were used for the densitometry normalization and analysis. Protein fold changes and availability of antibodies were taken into account for validation. ZIKV NS1 was probed to confirm the infection. **(D)** Classification of significantly dysregulated protein type at different time points after ZIKV infection. **(E)** The subcellular localizations of dysregulated proteins at different time points represented both quantitatively (left panel) and graphically (right panel).

We previously used a targeted aptamer-based approach to measure 1,305 ZIKV infection-induced host protein responses (Scaturro et al., 2018; Scaturro et al., 2019). Here, we performed a non-biased mass spectrometry (MS)-based proteomic study of ZIKV-infected Vero cells to extend and complement these previous studies. We identified a total of 7,455 cellular protein hits across all time points, with 6,443, 6,402, and 6,382 proteins identified at 12, 24, and 48 hpi, respectively (Supplementary Table S1). ZIKV proteins, including non-structural ones, also were identified in the virus-infected samples, but not in the mock samples, confirming infection status of the appropriate cells. Because the TMT analysis reports ZIKV:mock peptide and protein ratios, and ZIKV proteins are not expected in the mock samples, the ZIKV proteins are not considered hereafter. The number of significantly dysregulated cellular proteins was highest at 48 hpi, as earlier seen in our targeted studies (Scaturro et al., 2018; Scaturro et al., 2019) (Figure 1A and Tables 1, 2). Statistical tests indicated several thousand proteins were significantly dysregulated, although the vast majority had small fold-changes (Table 1). We, and many others, have previously applied fold-change cut-offs ranging from 1.5-fold to 2.0-fold for increased stringency, and we did likewise. Thus, nearly 400 proteins were significantly dysregulated using a 1.5-fold cut-off, which provided a rich source for subsequent bioinformatics analyses. Proteins dysregulated at a higher stringency of ≥2.0-fold are shown in Table 2. Less than 1% of the measured proteome underwent significant modifications within the first 24 h of infection. Most early dysregulated proteins were not significantly dysregulated at later time points, although the amount of MED13L (Mediator of RNA polymerase II transcription subunit 13-like) in ZIKV-infected cells was significantly higher than in mock cells at both early and late time points, but not significantly dysregulated at 24 hpi. We generated inter-time point heatmaps to facilitate interpretation of protein dysregulation patterns over the 72 h observation window and observed that except for MED13L, none of the significantly dysregulated proteins at either 12 or 24 hpi were significantly dysregulated in the same direction at later time points (Figures 1A,B). Several proteins (i.e., PLAUR, ARID5B, AHNAK, and ABLIM1) were significantly dysregulated in opposite directions across two time points (Figure 1B). Moreover, interaction networks obtained from protein data at 12 and 24 hpi identified 5 and 17 interacting molecules, respectively (Figure 1B, right panels), suggesting that these temporally altered proteins may interact with other biological process regulators during early phases of ZIKV infection. Immunoblots were performed to validate differential amounts of selected proteins at most time points (Figure 1C). Although there were differences in absolute dysregulation values, likely representing the different methods used, most protein dysregulations trended in the same direction. Enzymes and other unspecified molecule types were the most frequently observed proteins significantly differentially expressed at all time points investigated (Figure 1D). The majority of the regulated proteins at 12 hpi were mapped to the nucleus, while significantly altered proteins at 24 and 48 hpi mapped mainly to the cytoplasmic compartment (Figure 1E). This proteomic overview of dysregulated proteins indicated that ZIKV infection is capable of causing significant temporal alterations in the proteome of infected Vero cells.

**TABLE 1:** Numbers of ZIKV-induced significantly dysregulated proteins.

	**Total unique**	**12 h**	**24 h**	**48 h**
Number w/*p*-value < 0.05		72	393	2252
and F.C. > 1.05	2565	66	162	1094
and F.C. < 0.952		6	229	1156
and F.C. > 1.25	1355	27	44	534
and F.C. < 0.80		1	120	660
and F.C. > 1.33	855	20	29	317
and F.C. < 0.75		1	68	432
and F.C. > 1.50	374	10	13	120
and F.C. < 0.667		0	22	209
and F.C. > 2.00	78	4	6	21
and F.C. < 0.50		0	3	41
and F.C. > 2.50	30	1	4	7
and F.C. < 0.40		0	2	13

*Significance was determined by *T*-test and *Z*-Score as detailed in Materials & Methods. Specific proteins dysregulated ≥2.0-fold are listed in Table 2.*

**TABLE 2:** Vero cell proteins dysregulated ≥2.0-fold by ZIKV infection.

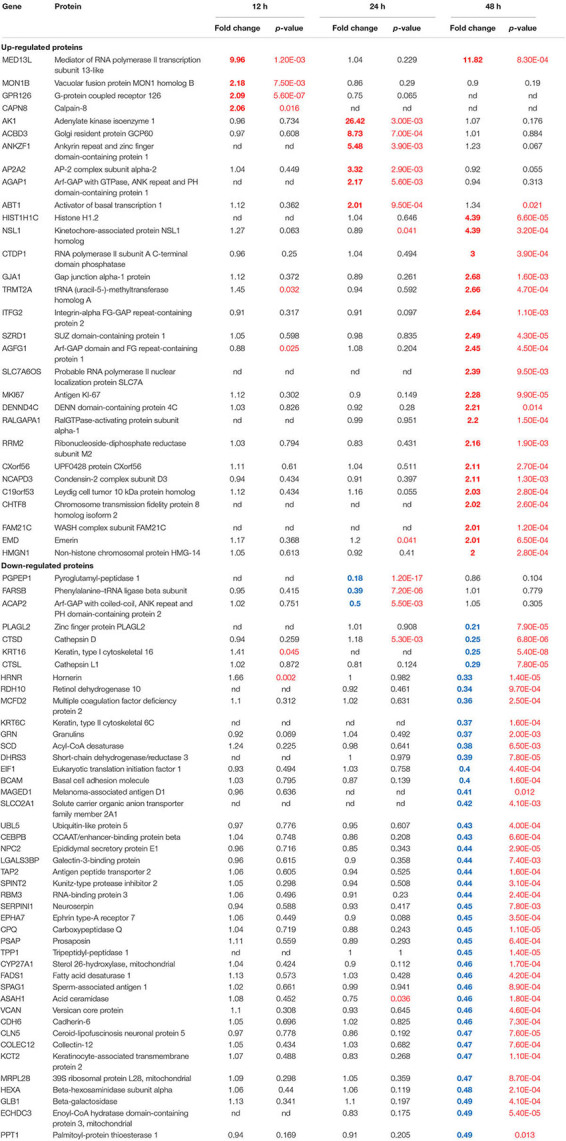

*Only proteins dysregulated ≥ 2.0-fold are listed. All proteins are listed in Supplementary Table S1. Entries are sorted, first by time, from 12 to 48 hpi, then by degree of dysregulation within each set. Values determined from three biological replicates. Proteins with significant *p*-value are red in *T*-Test column, those significantly up-regulated are shown in bold red and those significantly down-regulated are shown in bold blue. n.d. = not detected.*

### ZIKV Infection Dysregulates a Wide Variety of Bio-Functions and Pathways

**FIGURE 2:**
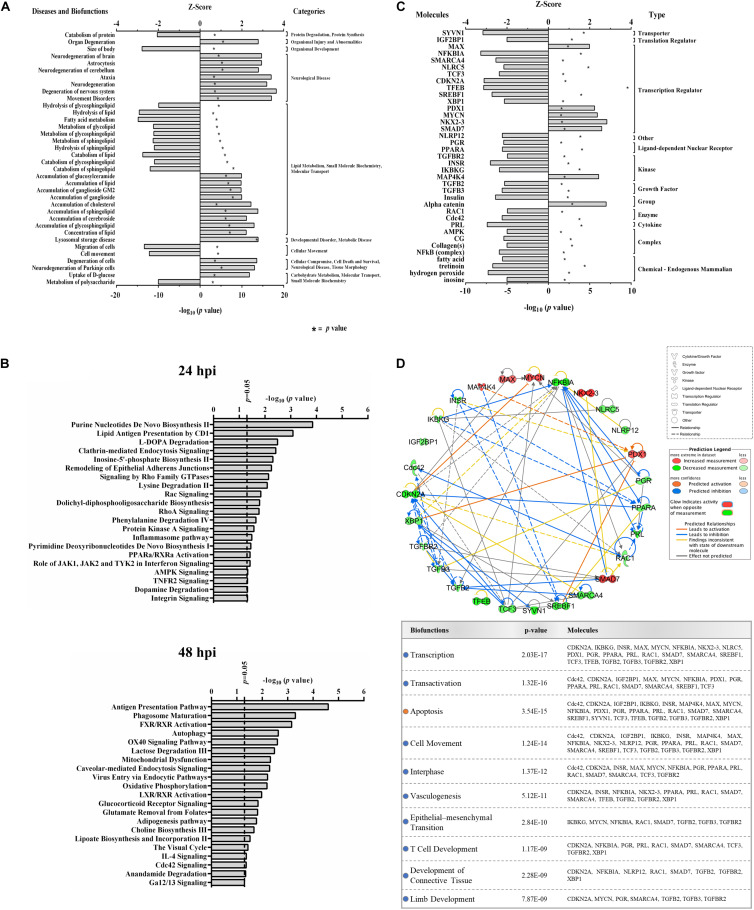
Proteomic prediction of top affected bio-functions, canonical pathways, and upstream molecules in Vero cells after ZIKV infection. **(A)** Predicted activation or inhibition patterns of top affected bio-functions at 48hpi based on *Z*-scores (upper *x*–axis) and log_10_ *p*–values (lower *x*–axis indicated by asterisk). Influenced bio-functions are grouped using major parental categories of functions (shown at right). **(B)** Prediction of top affected canonical pathways by ZIKV at 24 (upper panel) and 48 (lower panel) hpi. **(C)** Prediction of top affected upstream molecules and their possible activation or inhibition based on expression patterns of downstream proteins at 48 hpi. **(D)** Interaction network of top predicted upstream molecules (upper panel) and their related bio-functions (table in lower panel). Activation or inhibition patterns of connected bio-functions and interaction types of predicted molecules are measured according to *Z*-scores calculated from IPA software, which is based on the expression of differentially regulated downstream proteins at 48 hpi.

Functional analyses of dysregulated proteins identified bio-functions predicted to be highly affected by ZIKV infection. Due to the low number of modified proteins at early time points of ZIKV infection, IPA did not recognize any significant changes in bio-functions at 12 and 24 hpi. However, numerous functional alterations among a wide variety of bio-functions were predicted at 48 hpi. Among the largest number of affected bio-functions were protein alterations associated with neurological diseases and lipid metabolism (Figure 2A and Supplementary Table S2). Proteins linked to neurological abnormalities were predicted exclusively to be activated based on *Z*-score at 48 hpi, whereas proteins associated with lipid metabolism functions were almost equally divided into inhibited and activated functional states. Dysfunctions such as movement disorders, ataxia, neurodegeneration, astrocytosis, and lysosomal storage disease were among the top activated diseases (*Z*-scores ≥+2.7), and some functions including migration of cells, catabolism of lipid, size of body, hydrolysis of lipid, and fatty acid metabolism were among the top inhibited bio-functions (*Z*-scores ≤ −2.6) (Supplementary Table S2). The higher number of altered bio-functions and diseases involving lipid metabolism and neurological complications may suggest new links between the function of host cellular lipids and the development of ZIKV-induced cellular malfunctions. Analysis of the top 21 affected canonical pathways did not indicate commonalities across infection times, since entirely different sets of proteins were significantly dysregulated at each time point after ZIKV infection (Figure 2B). IPA could not establish confident inhibition or activation trends for ZIKV-modulated canonical pathways because there were too few ZIKV-modified pathway member proteins compared to background proteins. Nevertheless, with increasing duration of ZIKV infection, we observed more diversification of signaling pathways by 48 hpi compared to 24 hpi. Dysregulated pathways at 24 hpi were mainly assigned to metabolic processes, while pathways controlling immune responses, autophagy, endocytosis, phosphorylation, metabolism, visual phototransduction, cell movement, and others were among the top ZIKV-affected canonical pathways at 48 hpi. When compared with the first day of infection, the increasing diversity and specificity of altered pathways by 2 dpi indicates a dynamically evolving and increasingly complex host response during later stages of ZIKV infection that recruits and engages an increasing number of different cellular pathways.

IPA predicted 7 elevated activity functions (*Z*-scores ≥ + 1.97σ) and 30 elevated inhibitory function (*Z*-scores ≤ −1.96σ) (Figure 2C) from the lists of ZIKV-modulated proteins. About 35% of the affected upstream molecules were designated as regulators of transcription. One-third of these transcriptional regulators are predicted to be activated, whereas the remaining two-thirds are predicted to be inhibited by ZIKV. Interestingly, nuclear transcription factor NKX2-3 and NF-kappa-B inhibitor alpha (NFKBIA) were the most activated and inhibited factors, respectively. This suggests that ZIKV has the capacity to influence the host cell transcriptional machinery by coordinating different mechanisms of action. At least for NFKBIA, this has been linked to the regulation of NF kappa B mediated immunoregulatory and anti-inflammatory responses (Hamel et al., 2015). We interrogated the ability of affected upstream regulators to engage in an interconnecting network (Figure 2D). Most upstream regulators are predicted to interact with at least one other regulator molecule. NFKBIA and CDKN2A may serve as “master regulators” in this upstream network because they have the highest number of intermolecular interactions.

### ZIKV Alters Protein Interaction Networks Linked to Developmental Processes

**FIGURE 3:**
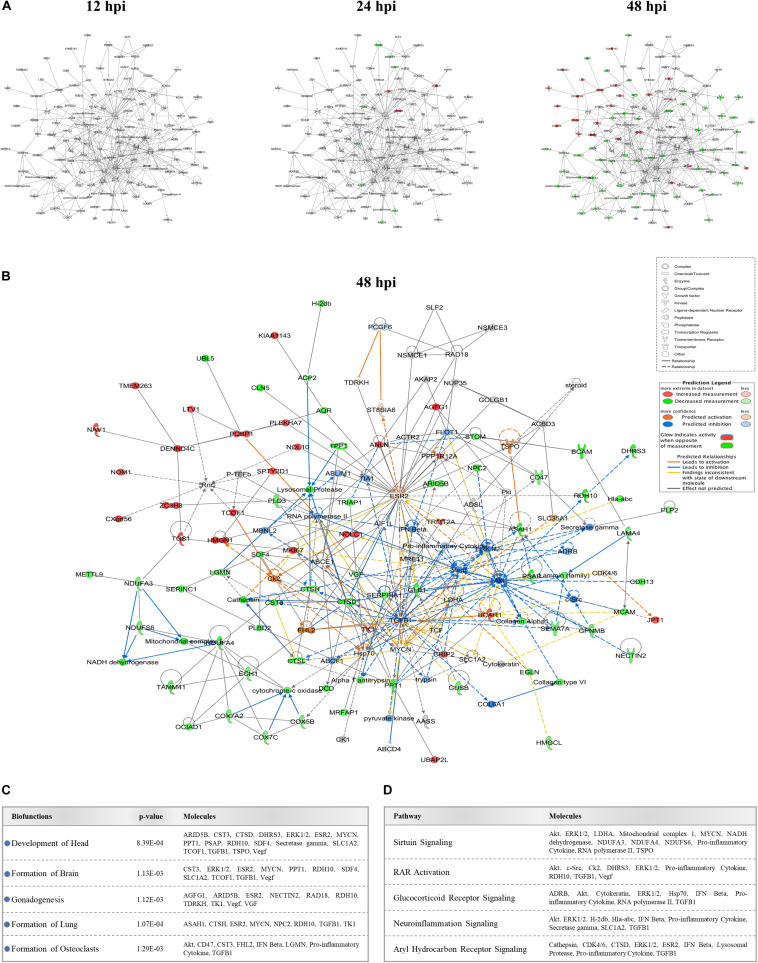
Effect of ZIKV infection on protein-protein interaction network of developmental abnormalities. **(A)** Alterations in the levels of proteins controlling developmental abnormalities network across three time-points. Higher and lower ZIKV-induced protein levels are represented in red and green, respectively; gray proteins denote that they were recognized in the present study but not significantly regulated; colorless proteins interact with molecules in the network but were not identified in our study. **(B)** IPA prediction of the protein regulation patterns that belong to developmental abnormality interaction networks but were either not differentially expressed or identified in our study. This prediction model was established through overlaying proteomic data at 48hpi onto the merged developmental abnormality network. Molecules shown in orange and blue represent predictions for activation and inhibition, respectively. **(C)** Selection of specific bio-functions that were predicted to be inhibited based on ZIKV-mediated changes in molecules regulating this network. **(D)** List of top-affected canonical pathways that could be connected to member proteins of developmental abnormalities network.

We identified several protein interaction networks comprising ≥20 focus molecules (*p*-score ≤ Log_*e*_ −30) that are associated with a broad spectrum of cellular functions, including lipid metabolism, inflammatory responses, and embryonic development. Those protein members of interaction networks with highly significant alterations corresponding to developmental disorders were selected and merged for temporal analysis (Figure 3A). No notable changes were detected at 12 hpi in this merged network. However, as time progressed to 48 hpi, significant increases and decreases in protein quantity patterns among interacting proteins indicated a complex and dynamic response profile of proteins associated with developmental processes in host cells exposed to ZIKV. The IPA prediction feature also suggested inhibition and activation of other member molecules in this network that were not experimentally identified by MS by 48 hpi (Figure 3B). TGFB1, Akt, ESR2, Vegf, ERK1/2, TK1, and Hsp70 were among the proteins with the largest number of intermolecular interactions in this network. We then connected the developmental disorder merged network to developmental phenotypes. Intriguingly, craniofacial and brain development scored among the highest, followed by gonad formation, lung development, and bone-resorbing osteoclast formation (Figure 3C). Pathway analyses of this network also revealed the involvement of several canonical biochemical pathways known to be important regulators of cellular differentiation, inflammatory processes, DNA repair, stress response, cell death, metabolism, and behavioral functions (Figure 3D).

### ZIKV Infection Targets Proteins Linked to Neurosensory Disorders

**FIGURE 4:**
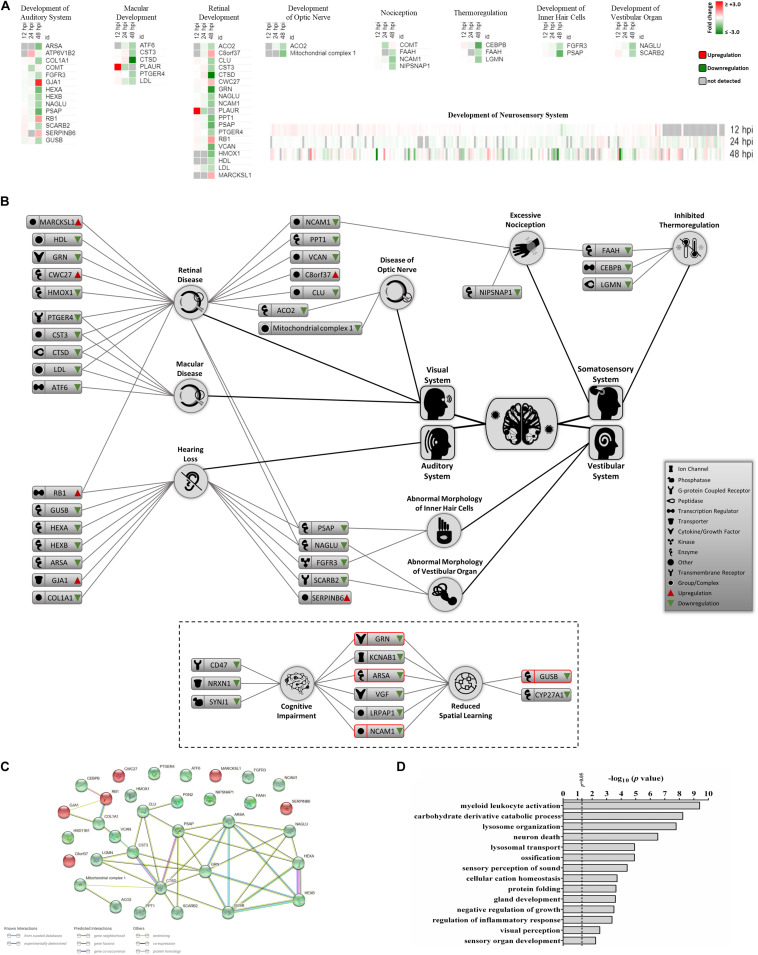
Proteomic prediction of ZIKV-induced changes in the neurosensory system. **(A)** Heatmaps of significantly dysregulated proteins involved in the development of neurosensory system. Most significant changes belonged to the 48 hpi dataset. Specific heatmaps that cover different subsets of neurosensory system are shown in upper panels. Lower panel represents the global overview heatmap for all proteins regulating the development of neurosensory system across three time-points from ZIKV-infected Vero cells. **(B)** Graphical representation of predicted ZIKV-triggered dysregulations in different parts of the neurosensory system by 2 dpi. Lower bordered panel indicates that a few proteins (ones with red margins) with influences on sensory nervous system together with some other differentially expressed proteins at 48 hpi could influence cognitive and learning functions based on IPA predictions. **(C)** Protein-protein interaction assessment of significantly regulated proteins causing neurosensory impairments based on their levels at 48 hpi. **(D)** Gene ontology analysis within the single class of biological process showing top affected pathways based on significantly dysregulated proteins with functions in neurosensory system at 48 hpi.

Recent clinical evidence suggests that ZIKV infection may cause neurosensory disorder development and cognitive impairments in children, even in those children that survived neonatal infection and do not show apparent complications (Garcez et al., 2016; Moore et al., 2017; Nielsen-Saines et al., 2019). Currently, no molecular study has attempted to link possible mediators and biomarkers of neurosensory diseases to ZIKV infection. We collated all known molecules related to the neurosensory system and its associated abnormalities and overlaid it with our proteomic data (Figure 4A). Significant ZIKV-mediated dysregulation was seen mostly at 48 hpi, impacting the levels of proteins controlling neurosensory system development. This included dysregulated biomarkers of neurosensory disorders in ZIKV infected host cells. A graphical illustration of molecules involved in ZIKV-induced impairment of the neurosensory system and their multiple functions in different neurosensory diseases is depicted in Figure 4B. A subset of proteins, including RB1, PSAP, NAGLU, FGFR3, SCARB2, LDL, CTSD, CST3, PTGER4, ACO2, NCAM1, and FAAH, were involved in at least two types of neurosensory dysfunctions. Moreover, proteins associated with cognitive impairments, like GRN, ARSA, NCAM1, and GUSB, also showed marked differences in protein levels (Figure 4B, lower panel). The largest numbers of identified dysregulated proteins were involved in retinal diseases and hearing loss. Bioinformatic assessment of all proteins corresponding to neurosensory diseases whose levels changed significantly between mock and infected showed that the vast majority of these proteins interacted with at least one other member (Figure 4C). Gene ontology analysis was also performed on this class of biological processes to provide an overview of most affected pathways (Figure 4D). In addition to auditory and visual perception and sensory organ development, we noted significant enrichment of other critical pathways that involve neurons, ossification, growth, gland development, and others. This highlights the complex functionality of ZIKV-affected host proteins and provides a first insight into molecular mechanisms and temporal events triggered by ZIKV infection that may drive the progression of neurosensory disorders and other developmental abnormalities. The protein members identified in this study and their affiliation with specific networks demonstrate, for the first time, an intricate relationship between ZIKV-induced host defenses and specific neuro-developmental processes and unveils novel mechanisms and pathways that qualify as potential new targets to combat ZIKV infection and downstream pathology.

## Discussion

Several groups have used proteomic approaches to elucidate ZIKV-triggered host protein responses (Beys-da-Silva et al., 2019; Rosa-Fernandes et al., 2019). Such studies were performed on different types of stem cells, non-neuronal and neuronal progenitors, lineages, and cell lines. These have been extremely informative in identifying signaling pathways, cellular processes controlled by ZIKV-affected host proteins, post-translational modifications, and protein–protein interactions (Berard et al., 2015; Beys-da-Silva et al., 2019; Hoxhaj et al., 2012; Rosa-Fernandes et al., 2019; Simon et al., 2015; Zahedi-Amiri et al., 2019). Most of these examined single time points post-infection. The lack of longitudinal data sets that provide profiles of time-specific protein dysregulation hampers our ability to differentiate ZIKV-induced persistent versus transient protein dysregulations (Araki and Milbrandt, 2003; Gangwani et al., 2005; Scaturro et al., 2018; Wen et al., 2019). Temporal protein expression monitoring data are critically important in predicting the potential impact specific protein changes within a dynamically changing proteome may have on certain signaling pathways and resulting bio-functions in infected cells. In addition, temporal proteomic patterns aid in the identification of key initiator proteins and proteomic networks that ZIKV uses to establish and maintain a successful infection cycle and, thus, contributes information relevant for clinical intervention. We recently used an aptamer-based targeted proteomic tool, SOMAscan, to examine the expression of >1,300 proteins across three separate time points in ZIKV-infected Vero and human U251 glioma cells. Similar to the current study, we identified a dynamic shift in proteomic changes from transient to persistent protein dysregulations, as ZIKV infection progressed over time (Scaturro et al., 2018; Scaturro et al., 2019). Here, we complemented our previous targeted studies by measuring about five times as many proteins in an unbiased manner, and, because recent clinical reports suggest a potential link between congenital ZIKV infection and neurosensory abnormalities in children that had suffered *in utero* ZIKV infection, we focused our analysis on proteins which showed significant dysregulation and were linked to the development of the neurosensory system.

Although most differentially expressed proteins at the early 12 hpi time point appeared to reflect acute responses to ZIKV infection and had returned to baseline at 48 hpi, almost one-third of the proteins identified at later times are linked to modulating neurosensory system functions and development. For example, E3 ubiquitin-protein ligase ZNRF2 is a zinc-finger protein that plays a role in protein polyubiquitination (McWhorter et al., 2003). In addition to its possible ubiquitin ligase activity to preserve neurotransmission and neuroplasticity, ZNRF2 is highly expressed during murine neuronal development (Rossoll et al., 2003). Some studies have reported ZIKV-mediated dysregulation of other E3 ubiquitin-protein ligases (Ahmad et al., 2012), but this is the first study implicating increases (>2-fold) in the amount of ZNRF2 in the early ZIKV-induced protein response at 12 hpi. Numerous other previously unreported zinc-finger proteins also were exclusively increased by ZIKV infection at the early time point, including ZNF830, ZSWIM8, ZPR1; a full list is shown in Figure 1B. ZPR1 may play roles in the development of the sensory nervous system and in embryonic growth. For example, Gangwani and colleagues used Zpr1^–/–^ murine blastocysts and showed that ZPR1 deficiency resulted in restricted proliferation of inner cell mass and abnormal trophectoderm formation, and lack of nuclear compartments like gem and Cajal bodies (Tunbridge et al., 2006). Absence of these sub-nuclear bodies leads to the mislocalization of survival motor neuron 1 (SMN1), a protein that interacts with ZPR1. SMN1 deficiency or dysfunction in motor neuron-like cells is associated with loss of growth cones, structural defects in axons that impair pathfinding and innervating abilities of these neurons, and contributes to Spinal Muscular Atrophy (SMA) pathogenesis (Allegri et al., 2010; Du et al., 2008; Tunbridge et al., 2006). We detected ZIKV-induced increased levels of ZPR1 at 12hpi in ZIKV-infected Vero cells. Ahmed et al. derived spinal cord neurons lacking SMN1 from a mice model of SMA and noticed that overexpression of ZPR1 in these neurons rescues abnormal axonal growth (Petrova et al., 2019). It is tempting to speculate that increases in ZPR1 protein during early stages of ZIKV infection reflects a protective host response, but this requires further studies.

Unlike the 12 hpi significantly differentially expressed proteins, all of which were found increased by ZIKV infection, there were significantly higher and lower ZIKV-induced protein levels at 24 hpi. Some proteins had been identified in previous transcriptomic analyses, but most had not previously been demonstrated at the proteomic level. Several of these proteins play key roles in neurosensory development. COMT (Catechol O-methyltransferase), a kinase reduced more than 1.5-fold at 24 hpi, is involved in neurosensory responses like nociception, balance, hearing, and catecholamine metabolism (Fernandez-Garcia et al., 2016; Yuan et al., 2015). Du and co-workers documented several severe malformations in a COMT missense murine model, including abnormal auditory startle response, impaired movement behavior, and loss of inner and outer hair cells with degeneration of cochlear neurons and vestibular defects, collectively causing deafness and degeneration of the vestibular sensory/balancing system in mice by 8 weeks of age (Menendez et al., 2017). Here we demonstrate ZIKV-mediated COMT reduction in Vero cells, providing first evidence for a potential molecular link between ZIKV infection and developmental impairment of cells within the auditory and vestibular sensory apparatus of the organ of Corti. Furthermore, levels of ATP6V1B2, a non-catalytic subunit of the peripheral V1 complex of vacuolar ATPase, were increased by 24 hpi. This protein is involved in the fusion of flaviviral and endosomal membranes (Wilcox et al., 2017; Yuan et al., 2014). Both ATP6V1B2 knockdown and over-expression in Zebrafish embryos resulted in loss of sensory hair cells, suggesting the critical role of this molecule during early development of the auditory system in vertebrates (Guo et al., 2015). Moreover, whole−exome sequencing of human clinical samples identified an ATP6V1B2 mutation that contributes to disrupted lysosome acidification and sensorineural hearing loss (Alfano et al., 2006; Kevany and Palczewski, 2010). Similar outcomes were observed with some DNA and RNA viruses, but this is the first report with ZIKV.

Proteomic host responses to ZIKV infection also affected proteins linked to the retinal sensory system. Levels of the transporter and ATP-binding protein ABCF1 were significantly reduced by 24 hpi. ABCF1 is an essential early gene in development and homozygous deletion leads to embryonic lethality of mice at day 3.5 post coitum (Powell et al., 2001). ABCF1 is involved in visual photo-transduction in the retina. Guo et al. discovered that ABCF1 is released from and binds to photoreceptor outer segments (POSs) to facilitate their phagocytosis by retinal pigment epithelial cells (RPECs) (Erkman et al., 2000). Photoreceptors in the retinal POS convert light into electrical signals, and the resulting photo-oxidative stress requires RPEC-modulated phagocytosis as an essential mechanism for recycling dysfunctional POSs to facilitate retinal regeneration (Etoh and Fukuda, 2015). Intriguingly, recent clinical evidence in children who had been exposed to ZIKV *in utero* demonstrated chorioretinal atrophy as one of several types of eye disorders observed in up to 7% of screened infants (Nielsen-Saines et al., 2019). No specific proteins or mechanisms have been proposed for ZIKV-associated retinal abnormalities. Our proteomic data implicate ABCF1 as a potential retinal target protein during ZIKV infection. If ZIKV were to infect retinal cells early during development, this may trigger ABCF1 (and/or related proteins) dysregulation in host cells, which disturbs retinal homeostasis at a critical time during development and contributes to long-term retinal abnormalities. Levels of PLAUR, a receptor for urokinase plasminogen activator, were significantly reduced at 24 hpi. PLAUR affects the migration, morphology, and the quantity of neurons. Reduced levels of PLAUR increases the susceptibility of RPECs to anoikis and causes decreased cellular movement (Powell et al., 2001; Zhao et al., 2019). Our observed ZIKV-induced decrease in PLAUR protein levels by 24 hpi raises the possibility that ZIKV can delay a developmental neural program that includes altered programmed cell death as shown in retinal cells, presumably resulting in the lack of metabolic support for photoreceptor cells, abnormal photoreceptor excitability, and retinal degeneration. Along the same lines, levels of ABLIM1, an actin-binding protein, were reduced by ZIKV infection by 24 hpi, despite its increase at later time points. Erkman and colleagues reported that ABLIM1 is required for proper pathfinding of retinal ganglion cells and axon guidance, because a dominant-negative mutation of this gene causes axons of the optic nerve to form abnormal trajectories to the optic disc and show defective fasciculation and growth (Erkman et al., 2000). Similarly, levels of the GTPase-activating protein ACAP2, which modulates neurite outgrowth (Etoh and Fukuda, 2015), also were reduced by 24 hpi. Additional proteins included AAAS, ACSF3, and ADSSL1, which are known to be associated with neurodevelopmental abnormalities that present with intellectual disability and impaired learning and memory. Collectively, our results identify a ZIKV-induced complex protein signature associated with neural development and cognitive deficits that is supported by clinical *in vivo* data linking congenital ZIKV infection with cognitive deficits in neonates (Nielsen-Saines et al., 2019; Zhao et al., 2019].

IPA predicted numerous elevated activity and inhibitory functions (Figure 2C). As indicated earlier, approximately one-third of the affected upstream molecules were transcription regulators, of which one-third are predicted to be activated and two-thirds are predicted to be inhibited by ZIKV. Many upstream regulators can be fit into an interconnecting network (Figure 2D) and most are predicted to interact with at least one other regulator molecule. The IPA “Grow” feature enabled us to link this interaction network to various bio-functions and diseases. In addition to activating apoptosis and inhibitory effects on transcription, transactivation, cell movement, and interphase, ZIKV infection was predicted to negatively impact upstream regulators associated with embryonic development. This included vasculogenesis, epithelial-mesenchymal transition, T cell development, development of connective tissue, and limb development (Figure 2D, lower panel).

To the best of our knowledge, the present proteomic study is the first to relate ZIKV-mediated temporal proteomic changes to clinical manifestations of neurosensory impairments identified in children with congenital exposure to ZIKV infection. We opted for proteomic profiling of temporal changes in protein expression in infected host cells as a promising way to discover early changes in protein networks and corresponding pathways that may initiate and promote downstream neurosensory defects. Temporal protein expression monitoring will also be helpful in determining cell-specific protein alterations in physiologically relevant cell, tissue, and animal models as they relate to neurosensory transduction. Additional *in vitro* and *in vivo* proteomic investigations in related models are warranted to better understand the impact ZIKV infection has on molecular mechanisms that initiate abnormalities in the development and function of the neurosensory system after ZIKV infection.

## Data Availability Statement

Spectra files in MGF format, the peptide identification results and the overall protein expression matrix are stored under accession MSV000085057 at the UCSD Centre for Computational Mass Spectrometry repository https://massive.ucsd.edu.

## Author Contributions

KG performed the most experimental work. YL performed all the MS analyses. KG, AZ-A, VS, TK, and KC analyzed all the data. KG and AZ-A drafted the manuscript. All authors edited and approved the final manuscript.

## Conflict of Interest

The authors declare that the research was conducted in the absence of any commercial or financial relationships that could be construed as a potential conflict of interest.

## References

[B1] Ahmad S. Wang Y. Shaik G. M. Burghes A. H. Gangwani L. (2012). The zinc finger protein ZPR1 is a potential modifier of spinal muscular atrophy. *Hum. Mol. Gen.* 21 2745–2758. 10.1093/hmg/dds102 22422766 PMC3363332

[B2] Alfano D. Iaccarino I. Stoppelli M. P. (2006). Urokinase signaling through its receptor protects against anoikis by increasing BCL-xL expression levels. *J. Biol. Chem.* 281 17758–17767. 10.1074/jbc.m601812200 16632475

[B3] Allegri M. Gregori M. De Niebel T. Minella C. Tinelli C. Govoni S. (2010). Pharmacogenetics and postoperative pain: a new approach to improve acute pain management. *Minerva Anestesiol.* 76 937–944. 21102389

[B4] Araki T. Milbrandt J. (2003). ZNRF proteins constitute a family of presynaptic E3 ubiquitin ligases. *J. Neurosci.* 23 9385–9394. 10.1523/jneurosci.23-28-09385.2003 14561866 PMC6740566

[B5] Berard A. R. Coombs K. M. Severini A. (2015). Quantification of the host response proteome after herpes simplex virus type 1 infection. *J. Proteome Res.* 14 2121–2142. 10.1021/pr5012284 25815715

[B6] Besnard M. Eyrolle-Guignot D. Guillemette-Artur P. Lastere S. Bost-Bezeaud F. Marcelis L. (2016). Congenital cerebral malformations and dysfunction in fetuses and newborns following the 2013 to 2014 Zika virus epidemic in French Polynesia. *Eurosurveillance* 21:30181. 10.2807/1560-7917.ES.2016.21.13.30181 27063794

[B7] Beys-da-Silva W. O. Rosa R. L. Santi L. Berger M. Park S. K. Campos A. R. (2019). Zika virus infection of human mesenchymal stem cells promotes differential expression of proteins linked to several neurological diseases. *Mol. Neurobiol.* 56 4708–4717. 10.1007/s12035-018-1417-x 30377986 PMC6491274

[B8] Brasil P. Pereira J. P. Jr. Moreira M. E. Nogueira R.M. Ribeiro Damasceno L. Wakimoto M. (2016). Zika virus infection in pregnant women in Rio de Janeiro. *New Eng. J. Med.* 375 2321–2334. 26943629 10.1056/NEJMoa1602412 PMC5323261

[B9] Calvet G. Aguiar R. S. Melo A. S. O. Sampaio S. A. de Filippis I. Fabri A. (2016). Detection and sequencing of Zika virus from amniotic fluid of fetuses with microcephaly in Brazil: a case study. *Lancet Infect. Dis.* 16 653–660. 10.1016/s1473-3099(16)00095-5 26897108

[B10] Du X. Schwander M. Moresco E. M. Viviani P. Haller C. Hildebrand M. S. (2008). A catechol-O-methyltransferase that is essential for auditory function in mice and humans. *Proc. Natl. Acad. Sci. U.S.A.* 105 14609–14614. 10.1073/pnas.0807219105 18794526 PMC2567147

[B11] Erkman L. Yates P. A. McLaughlin T. McEvilly R. J. Whisenhunt T. O‘Connell S. H. (2000). A POU domain transcription factor–dependent program regulates axon pathfinding in the vertebrate visual system. *Neuron* 28 779–792. 10.1016/s0896-6273(00)00153-7 11163266

[B12] Etoh K. Fukuda M. (2015). Structure-function analyses of the small GTPase Rab35 and its effector protein centaurin-β2/ACAP2 during neurite outgrowth of PC12 cells. *J. Biol. Chem.* 290 9064–9074. 10.1074/jbc.m114.611301 25694427 PMC4423693

[B13] Fernandez-Garcia M. D. Meertens L. Chazal M. Hafirassou M. L. Dejarnac O. Zamborlini A. (2016). Vaccine and wild-type strains of yellow fever virus engage distinct entry mechanisms and differentially stimulate antiviral immune responses. *mBio* 7:e01956-15. 10.1128/mBio.01956-15 PMC4752603 26861019

[B14] Gangwani L. Flavell R. A. Davis R. J. (2005). ZPR1 is essential for survival and is required for localization of the survival motor neurons (SMN) protein to Cajal bodies. *Mol. Cell. Biol.* 25 2744–2756. 10.1128/mcb.25.7.2744-2756.2005 15767679 PMC1061650

[B15] Garcez P. P. Loiola E. C. da R. Madeiro Costa L.M. Higa Trindade P. Delvecchio R. (2016). Zika virus impairs growth in human neurospheres and brain organoids. *Science* 352 816–818. 10.1126/science.aaf6116 27064148

[B16] Glover K. K. Gao A. Zahedi-Amiri A. Coombs K. M. (2019). Vero cell proteomic changes induced by Zika virus infection. *Proteomics* 19:e1800309. 10.1002/pmic.201800309 30578658

[B17] Guo F. Ding Y. Caberoy N. Alvarado G. Wang F. Chen R. (2015). ABCF1 extrinsically regulates retinal pigment epithelial cell phagocytosis. *Mol. Biol. Cell* 26 2311–2320. 10.1091/mbc.e14-09-1343 25904329 PMC4462947

[B18] Hamel R. Dejarnac O. Wichit S. Ekchariyawat P. Neyret A. Luplertlop N. (2015). Biology of Zika virus infection in human skin cells. *J. Virol.* 89 8880–8896. 26085147 10.1128/JVI.00354-15 PMC4524089

[B19] Honein M. A. Dawson A. L. Petersen E. E. Jones A. M. Lee E. H. Yazdy M. M. (2017). Birth defects among fetuses and infants of US women with evidence of possible Zika virus infection during pregnancy. *JAMA* 317 59–68. 27960197 10.1001/jama.2016.19006

[B20] Hoxhaj G. Najafov A. Toth R. Campbell D. G. Prescott A. R. MacKintosh C. (2012). ZNRF2 is released from membranes by growth factors and, together with ZNRF1, regulates the Na+/K+ ATPase. *J. Cell Sci.* 125 4662–4675. 10.1242/jcs.110296 22797923 PMC3500867

[B21] Kevany B. M. Palczewski K. (2010). Phagocytosis of retinal rod and cone photoreceptors. *Physiology* 25 8–15. 10.1152/physiol.00038.2009 20134024 PMC2839896

[B22] Lopes Moreira M. E. Nielsen-Saines K. Brasil P. Kerin T. Damasceno L. Pone M. (2018). Neurodevelopment in infants exposed to Zika virus in utero. *New Engl. J. Med.* 379 2377–2379. 30575464 10.1056/NEJMc1800098 PMC6478167

[B23] Madaschi V. Mecca T. P. de Macedo E. C. Paula C. S. (2016). Bayley-III scales of infant and toddler development: transcultural adaptation and psychometric properties. *Paidéia* 26 189–197. 10.1590/1982-43272664201606

[B24] Martines R. B. Bhatnagar J. Keating M. K. Silva-Flannery L. Muehlenbachs A. Gary J. (2016). Notes from the field: evidence of Zika virus infection in brain and placental tissues from two congenitally infected newborns and two fetal losses—Brazil, 2015. *MMWR* 65 159–160. 10.15585/mmwr.mm6506e1 26890059

[B25] McWhorter M. L. Monani U. R. Burghes A. H. Beattie C. E. (2003). Knockdown of the survival motor neuron (Smn) protein in zebrafish causes defects in motor axon outgrowth and pathfinding. *J. Cell Biol.* 162 919–932. 10.1083/jcb.200303168 12952942 PMC1761110

[B26] Menendez I. Carranza C. Herrera M. Marroquin N. Foster J. Cengiz F. B. (2017). Dominant deafness–onychodystrophy syndrome caused by an ATP6V1B2 mutation. *Clin. Case Rep.* 5 376–379. 10.1002/ccr3.761 28396750 PMC5378843

[B27] Moore C. A. Staples J. E. Dobyns W. B. Pessoa A. Ventura C. V. Fonseca E. B. (2017). Characterizing the pattern of anomalies in congenital Zika syndrome for pediatric clinicians. *JAMA Peds.* 171 288–295. 10.1001/jamapediatrics.2016.3982 PMC5561417 27812690

[B28] Nielsen-Saines K. Brasil P. Kerin T. Vasconcelos Z. Gabaglia C. R. Damasceno L. (2019). Delayed childhood neurodevelopment and neurosensory alterations in the second year of life in a prospective cohort of ZIKV-exposed children. *Nature Med.* 25 1213–1217. 10.1038/s41591-019-0496-1 31285631 PMC6689256

[B29] Petrova E. Gracias S. Beauclair G. Tangy F. Jouvenet N. (2019). Uncovering flavivirus host dependency factors through a genome-wide gain-of-function screen. *Viruses* 11:68. 10.3390/v11010068 30650657 PMC6356745

[B30] Powell E. M. Mars W. M. Levitt P. (2001). Hepatocyte growth factor/scatter factor is a motogen for interneurons migrating from the ventral to dorsal telencephalon. *Neuron* 30 79–89. 10.1016/s0896-6273(01)00264-1 11343646

[B31] Rosa-Fernandes L. Cugola F. R. Russo F. B. Kawahara R. de Melo Freire C. C. Leite P. E. C. (2019). Zika virus impairs neurogenesis and synaptogenesis pathways in human neural stem cells and neurons. *Front. Cell. Neurosci.* 13:64. 10.3389/fncel.2019.00064 30949028 PMC6436085

[B32] Rossoll W. Jablonka S. Andreassi C. Kröning A. K. Karle K. Monani U. R. (2003). Smn, the spinal muscular atrophy–determining gene product, modulates axon growth and localization of β-actin mRNA in growth cones of motoneurons. *J. Cell Biol.* 163 801–812. 10.1083/jcb.200304128 14623865 PMC2173668

[B33] Sarno M. Sacramento G. A. Khouri R. do M. S. Rosario F. Costa Archanjo G. (2016). Zika virus infection and stillbirths: a case of hydrops fetalis, hydranencephaly and fetal demise. *PLoS Neg. Trop. Dis.* 10:e0004517. 10.1371/journal.pntd.0004517 26914330 PMC4767410

[B34] Scaturro P. Kastner A. L. Pichlmair A. (2019). Chasing intracellular Zika virus using proteomics. *Viruses* 11:878. 10.3390/v11090878 31546825 PMC6783930

[B35] Scaturro P. Stukalov A. Haas D. A. Cortese M. Draganova K. Płaszczyca A. (2018). An orthogonal proteomic survey uncovers novel Zika virus host factors. *Nature* 561 253–257. 10.1038/s41586-018-0484-5 30177828

[B36] Scherer D. C. Brockman J. A. Chen Z. Maniatis T. Ballard D. W. (1995). Signal-induced degradation of I-Kappa-B-Alpha requires site-specific ubiquitination. *Proc. Natl. Acad. Sci. U.S.A.* 92 11259–11263. 10.1073/pnas.92.24.11259 7479976 PMC40611

[B37] Schuler-Faccini L. Ribeiro E. M. Feitosa I. M. Horovitz D. D. Cavalcanti D. P. Pessoa A. (2016). Possible association between Zika virus infection and microcephaly—Brazil, 2015. *MMWR* 65 59–62. 26820244 10.15585/mmwr.mm6503e2

[B38] Sher A. Glover K. Coombs K. M. (2019). Zika virus infection disrupts astrocytic proteins involved in synapse control and axon guidance. *Front. Microbiol.* 10:596. 10.3389/fmicb.2019.00596 30984137 PMC6448030

[B39] Sielaff M. Kuharev J. Bohn T. Hahlbrock J. Bopp T. Tenzer S. (2017). Evaluation of FASP, SP3, and iST protocols for proteomic sample preparation in the low microgram range. *J. Proteome Res.* 16 4060–4072. 10.1021/acs.jproteome.7b00433 28948796

[B40] Simon P. F. McCorrister S. Hu P. Chong P. Silaghi A. Westmacott G. (2015). Highly pathogenic H5N1 and novel H7N9 influenza A viruses induce more profound proteomic host responses than seasonal and pandemic H1N1 strains. *J. Proteome Res.* 14 4511–4523. 10.1021/acs.jproteome.5b00196 26381135

[B41] Tunbridge E. M. Harrison P. J. Weinberger D. R. (2006). Catechol-o-methyltransferase, cognition, and psychosis: Val158Met and beyond. *Biol. Psychol.* 60 141–151. 10.1016/j.biopsych.2005.10.024 16476412

[B42] Wen F. Armstrong N. Hou W. Cruz-Cosme R. Obwolo L. A. Ishizuka K. (2019). Zika virus increases mind bomb 1 levels, causing degradation of pericentriolar material 1 (PCM1) and dispersion of PCM1 granules from the centrosome. *J. Biol. Chem.* 294 18742–18755. 10.1074/jbc.ra119.010973 31666336 PMC6901299

[B43] Wilcox S. M. Arora H. Munro L. Xin J. Fenninger F. Johnson L. A. (2017). The role of the innate immune response regulatory gene ABCF1 in mammalian embryogenesis and development. *PLoS One* 12:e0175918. 10.1371/journal.pone.0175918 28542262 PMC5438103

[B44] Yuan Y. Gao X. Xin F. Dai P. (2015). Atp6v1b2 plays important roles in the early development of hearing, the pectoral fin, the cardiovascular system, and the swim bladder of the Zebrafish, supporting a role for the gene in syndromic hearing loss. *bioRxiv [Preprint]* 10.1101/024240

[B45] Yuan Y. Zhang J. Chang Q. Zeng J. Xin F. Wang J. (2014). De novo mutation in ATP6V1B2 impairs lysosome acidification and causes dominant deafness-onychodystrophy syndrome. *Cell Res.* 24 1370–1373. 10.1038/cr.2014.77 24913193 PMC4220163

[B46] Zahedi-Amiri A. Sequiera G. L. Dhingra S. Coombs K. M. (2019). Influenza a virus-triggered autophagy decreases the pluripotency of human-induced pluripotent stem cells. *Cell Death Dis.* 10:337. 10.1038/s41419-019-1567-4 PMC6472374 31000695

[B47] Zhao Z. Shang Z. de Vasconcelos Z. F. M. Li C. Jiang Y. Zu S. (2019). Of mice and children: Zika virus infection leads to variable defects in multiple neurological functions and behaviors. *SSRN Electr. J.* 10.2139/ssrn.3335070 PMC7509663 32999822

