# Supplementary figures and images for: Zika Infection Disrupts Proteins Involved in the Neurosensory System
Source: Front Cell Dev Biol. 2020 Jul 29;8:571. doi: 10.3389/fcell.2020.00571 (PMC7403212; doi:10.3389/fcell.2020.00571)

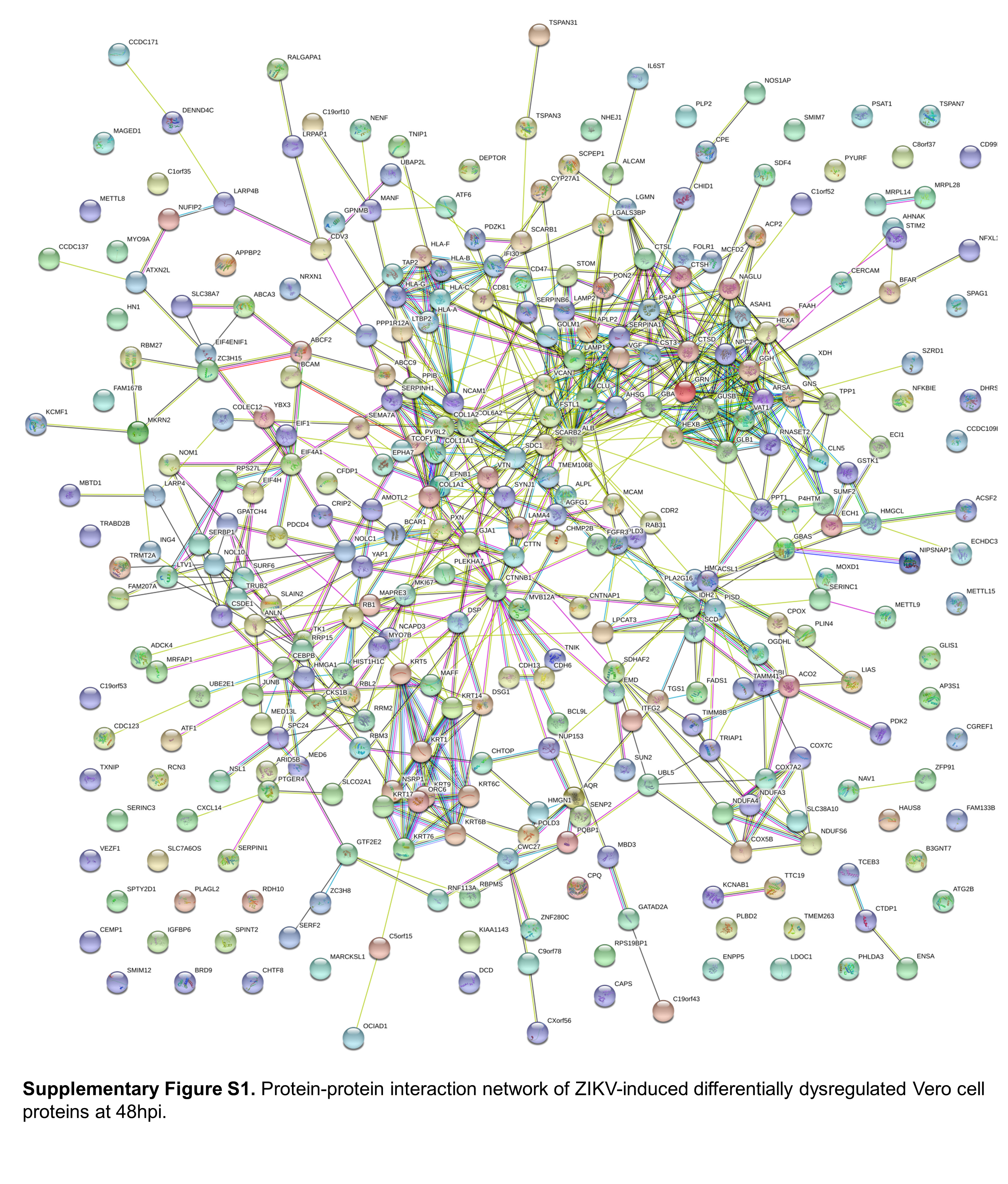

Supplement: Supplementary file 1 [file Image_1.TIF]
